# Understanding the Molecular Basis of the Multiple Mitochondrial Dysfunctions Syndrome 2: The Disease-Causing His96Arg Mutation of BOLA3

**DOI:** 10.3390/ijms241411734

**Published:** 2023-07-21

**Authors:** Beatrice Bargagna, Lucia Banci, Francesca Camponeschi

**Affiliations:** 1 Department of Chemistry, University of Florence, Via Della Lastruccia 3, Sesto Fiorentino, 50019 Florence, Italy; bargagna@cerm.unifi.it; 2 Magnetic Resonance Center CERM, University of Florence, Via Luigi Sacconi 6, Sesto Fiorentino, 50019 Florence, Italy; 3 Consorzio Interuniversitario Risonanze Magnetiche di Metalloproteine (CIRMMP), Via Luigi Sacconi 6, Sesto Fiorentino, 50019 Florence, Italy

**Keywords:** BOLA3, GLRX5, MMDS2, multiple mitochondrial dysfunctions syndrome, iron-sulfur clusters, iron-sulfur cluster biogenesis, ISC machinery, mitochondria

## Abstract

Multiple mitochondrial dysfunctions syndrome type 2 with hyperglycinemia (MMDS2) is a severe disorder of mitochondrial energy metabolism, associated with biallelic mutations in the gene encoding for BOLA3, a protein with a not yet completely understood role in iron-sulfur (Fe-S) cluster biogenesis, but essential for the maturation of mitochondrial [4Fe-4S] proteins. To better understand the role of BOLA3 in MMDS2, we have investigated the impact of the p.His96Arg (c.287A > G) point mutation, which involves a highly conserved residue, previously identified as a [2Fe-2S] cluster ligand in the BOLA3-[2Fe-2S]-GLRX5 heterocomplex, on the structural and functional properties of BOLA3 protein. The His96Arg mutation has been associated with a severe MMDS2 phenotype, characterized by defects in the activity of mitochondrial respiratory complexes and lipoic acid-dependent enzymes. Size exclusion chromatography, NMR, UV-visible, circular dichroism, and EPR spectroscopy characterization have shown that the His96Arg mutation does not impair the interaction of BOLA3 with its protein partner GLRX5, but leads to the formation of an aberrant BOLA3-[2Fe-2S]-GLRX5 heterocomplex, that is not functional anymore in the assembly of a [4Fe-4S] cluster on NFU1. These results allowed us to rationalize the severe phenotype observed in MMDS2 caused by His96Arg mutation.

## 1. Introduction

Mitochondria play essential roles in numerous processes in human cells, ranging from ATP production, different metabolic pathways, the biosynthesis of amino acids, lipids, and essential protein cofactors such as iron-sulfur (Fe-S) clusters [1,2]. The latter performs a multiplicity of functions in cells, such as electron transport, enzymatic catalysis, DNA maintenance, and gene expression regulation [3,4,5]. The synthesis of Fe-S clusters in the mitochondria of human cells is tightly controlled by the Iron-Sulfur Cluster assembly machinery (ISC machinery hereafter) [6,7,8], which performs the biogenesis of numerous Fe-S cluster containing proteins involved in essential processes such as the respiration and the Krebs Cycle [9,10]. Hence, the activity of mitochondria strictly relies on the correct functioning of the ISC machinery, and several mutations in the genes encoding for many of its components have been connected to several rare but severe diseases in humans. In particular, mutations in the genes encoding for the proteins acting late in the ISC machinery and responsible for the maturation of [4Fe-4S] proteins, have been connected to a group of autosomal recessive mitochondrial energy metabolism disorders, called multiple mitochondrial dysfunctions syndromes (MMDSs) types 1 to 5, associated with NFU1, BOLA3, IBA57, ISCA2, and ISCA1, respectively [11,12,13,14,15].

These disorders present early in life with severe neurodegeneration, weakness, respiratory failure, lactic acidosis, and hyperglycinemia, leading to death in the first year of life [11]. Although the cellular function of many of the proteins associated with MMDSs is only partially understood, the majority of the MMDSs phenotypes arise from defects in the maturation of mitochondrial [4Fe-4S] proteins, resulting in a reduced or impaired activity of mitochondrial respiratory complexes and lipoic acid-dependent enzymes, such as pyruvate dehydrogenase (PDH), 2-ketoglutarate dehydrogenase (KGDH) and protein H of the Glycine Cleavage System (GCS) [16].

MMDS type 2 (MMDS2; MIM#614299) is caused by mutations in BOLA3, a protein required in humans for the assembly of mitochondrial [4Fe-4S]-proteins, but with a not yet completely understood function [17]. BOLA3 interacts with GLRX5 [18,19], forming a hetero-dimeric complex that binds a [2Fe-2S]^2+^ cluster bridged between the two proteins. The BOLA3-[2Fe-2S]-GLRX5 complex acts in vitro as a [2Fe-2S]-cluster chaperone for several mitochondrial partners [20,21]. One of these is NFU1, a protein involved in the final steps of the maturation of mitochondrial [4Fe-4S] cluster-binding enzymes [17,22], which receives in vitro two [2Fe-2S]^2+^ clusters from two BOLA3-[2Fe-2S]-GLRX5 complexes. The two [2Fe-2S]^2+^ clusters are reductively coupled to form a [4Fe-4S]^2+^ on NFU1 [21]. This pathway, which was proposed to be an alternative to the pathway involving the [4Fe-4S] cluster transfer from the ISCA1-ISCA2 complex to NFU1 and to be exclusively activated under oxidative cellular conditions [21], connects BOLA3 to the maturation of mitochondrial [4Fe-4S] proteins and might be altered in MMDS2.

Here we investigated the H96R BOLA3 pathogenic mutant, with the aim of understanding the molecular basis of its role in MMDS2 and further elucidating the cellular function of BOLA3 and its involvement in the maturation of [4Fe-4S] clusters in mitochondria. The His96Arg mutation was found in the Japanese population and caused severe lactic acidosis and combined respiratory chain complex deficiencies due to decreased complex I, II, and IV activity, multiple organ failure, and hypertrophic cardiomyopathy seizures. High lactate and glycine levels were found in the majority of the patients, who died in the first year of life [23,24,25].

We applied various spectroscopic and biochemical techniques, including NMR, EPR, UV-Visible (UV-Vis), circular dichroism (CD) spectroscopy, and analytical size exclusion chromatography (SEC) to investigate the impact of the His96Arg mutation on the structure of BOLA3 and its interaction with GLRX5. Moreover, we investigated the effects of the mutation on the cluster binding properties of BOLA3 and the subsequent formation of the hetero-dimeric BOLA3-[2Fe-2S]-GLRX5 complex. Finally, we investigated the effect of the mutation on the proposed function of BOLA3 in assisting the [4Fe-4S] cluster assembly on NFU1. We found that the His96Arg mutation does not affect the overall structure and folding of BOLA3, nor the interaction with its partner protein GLRX5. However, we found that the mutation, although still allowing the binding of a bridged [2Fe-2S]^2+^ cluster in the H96R BOLA3-[2Fe-2S]-GLRX5 hetero-complex, severely affects the ability of such complex to act as a [2Fe-2S] cluster chaperone for NFU1, thus allowing us to rationalize the phenotype observed for the His96Arg pathogenic mutation of BOLA3 protein in MMDS2.

## 2. Results

### 2.1. The His96Arg Substitution Does Not Affect the Structure of BOLA3

**Figure 1:**
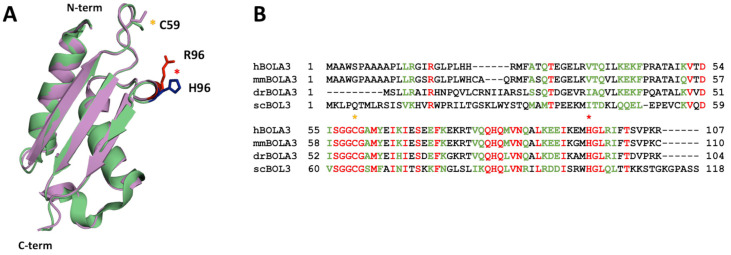
His96Arg mutation involves a highly conserved residue of BOLA3. (**A**) Superimposition of the solution structure of WT BOLA3 (green, PDB: 2NCL, [19]) and of the putative structure of H96R BOLA3 (violet, see Section 4). The histidine at position 96 in the wild-type protein, which is mutated to arginine in MMDS2, is colored blue on the ribbon structure of WT BOLA3. The pathogenic Arg96 mutation is shown in red on the ribbon structure of H96R BOLA3. (**B**) Multiple sequence alignment of BOLA3 with BolA homologues in several model organisms using Clustal Omega [26]. h = *Homo sapiens*; mm = *Mus musculus*; dr = *Danio rerio*; sc = *Saccharomyces cerevisiae*. Identical residues are colored in red, and similar residues are shown in green. Red and yellow asterisks in (**A**,**B**) indicate the location of conserved Cys59 and His96 [2Fe-2S] cluster ligands in yeast human BOLA3, respectively.

Structural modeling of the His96Arg variant on the solution structure of human BOLA3 (PDB: 2NCL, [19]) showed that the substitution is located in a flexible loop region of the protein and is highly solvent exposed, and thus not in contact with other protein regions (Figure 1A).

Although the substitution involves a C-terminal histidine residue which is fully conserved across BolA-like proteins from eukaryotic species (Figure 1B), no changes in the structure of the H96R BOLA3 are observed as the mutated residue is located in a flexible loop and therefore its change is not expected to determine any structural change.

**Figure 2:**
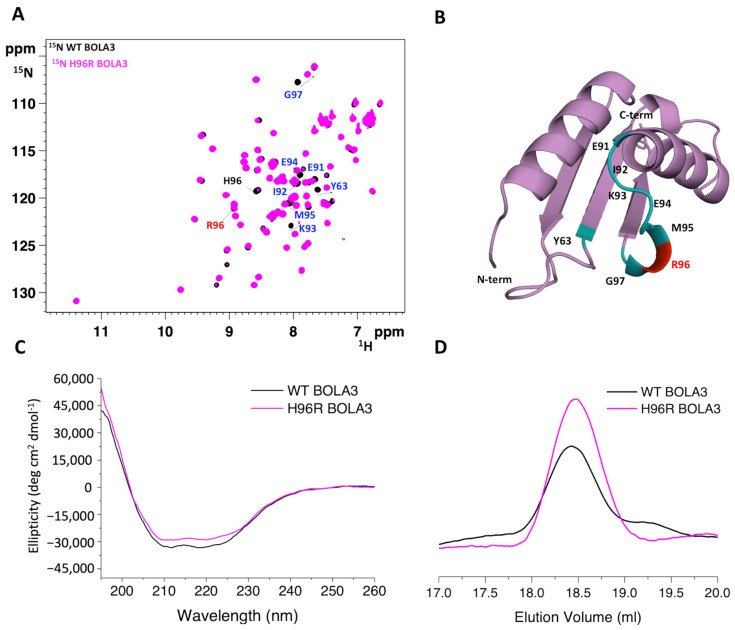
The His96Arg mutation does not affect the overall fold of BOLA3 protein. (**A**) Superimposition of the ^1^H-^15^N HSQC spectra of ^15^N-labeled H96R BOLA3 (magenta) and ^15^N-labeled WT BOLA3 (black), acquired at 500 MHz at 298 K; (**B**) mapping of the chemical shift changes (shown in cyan) due to the His96Arg mutation on the model structure of H96R BOLA3. Far-UV CD spectra (**C**) and analytical SEC (**D**) of WT BOLA3 (black) and H96R BOLA3 (magenta). The model shown in panel 2B was rendered with Pymol 2.3.5.

The ^1^H-^15^N HSQC NMR spectrum of ^15^N-labeled H96R BOLA3 is indicative of a well-folded protein, with the NH cross-peaks having wide dispersions in the ^1^H dimension and sharp linewidths (Figure 2A). The spectrum is well superimposable to that of ^15^N-labeled WT BOLA3. Small chemical shift changes were observed only for residues of the loop containing the His96Arg mutation and residues nearby (Figure 2B). Accordingly, CD spectra showed high similarity between H96R BOLA3 and WT BOLA3 secondary structure content (Figure 2C), thus experimentally confirming that the mutation of His96 into Arg does not affect the secondary structure of the protein.

The quaternary structure and the stability of the H96R BOLA3 mutant were investigated via analytical size exclusion chromatography (SEC) and variable temperature CD (VTCD), by comparing the elution and the melting profiles, respectively, of the H96R BOLA3 mutant with those of the WT BOLA3 protein. The two proteins were eluted with a single peak at the same elution volume (~18.4 mL, Figure 2D), indicating that the H96R mutant and the WT BOLA3 protein are monomeric in solution [18]. H96R BOLA3 variant showed a VTCD trace very similar to that of WT BOLA3, with very similar melting temperature (Figure S1A,B), indicating that the two proteins have essentially the same stability.

Overall, these results indicate that the substitution of histidine in position 96 with a positively charged arginine only perturbs the local environment of the loop containing the mutation, with no significant modification of the protein folding, quaternary structure, and stability.

### 2.2. The His96Arg Substitution Does Not Impair the Interaction between BOLA3 and GLRX5

Although the exact cellular function of the BOLA3 protein is still elusive, several in vitro and in vivo studies showed that the BOLA3-GLRX5 hetero-dimeric complex can function as a [2Fe-2S] cluster chaperone in the ISC machinery [18,21]. We, therefore, investigated whether the His96Arg substitution could affect the interaction of BOLA3 with GLRX5.

**Figure 3:**
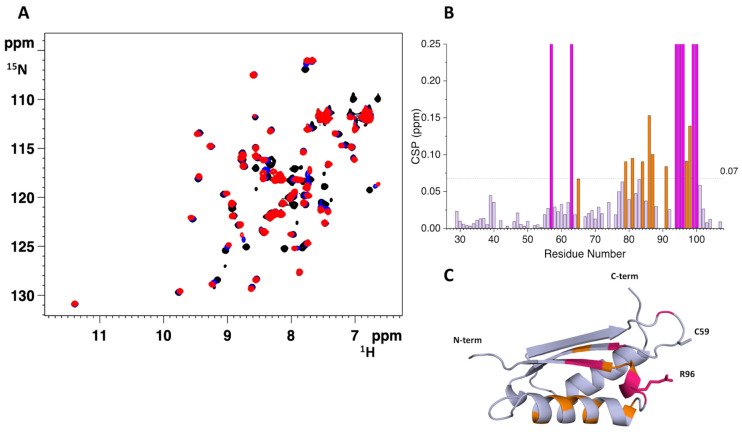
The His96Arg mutation does not impact the formation of the apo H96R BOLA3-GLRX5 complex. (**A**) Superimposition of the ^1^H-^15^N HSQC spectra of ^15^N-labeled H96R BOLA3 in the absence (black) and the presence of 0.5 eq. (blue) and 1.0 eq. (red) of apo unlabeled GLRX5, acquired at 500 MHz and 298 K; (**B**) chemical shift perturbations of ^15^N-labeled H96R BOLA3 before and after the addition of 1.0 eq. of unlabeled GLRX5, calculated as (((ΔH)^2^ + (ΔN/5)^2^)/2)^1/2^. The indicated threshold value (obtained by averaging CSP values plus 1σ) were used to define meaningful chemical shift differences. (**C**) Mapping of the meaningful chemical shift changes due to the interaction with GLRX5 on the model structure of H96R BOLA3. Residues experiencing chemical shift variation are shown in orange, while residues experiencing line-broadening are shown in magenta in both (**B**,**C**) panels. The model shown in panel 3C was rendered with Pymol 2.3.5.

Either apo ^15^N-labeled H96R BOLA3 or apo ^15^N-labeled GLRX5 were titrated with unlabeled apo GLRX5 or apo H96R BOLA3, respectively, and the observed spectral changes in ^1^H-^15^N HSQC spectra were quantified and mapped on the structural model of H96R BOLA3 and the solution structure of GSH-bound apo GLRX5 (PDB: 2MMZ, [27]). Several chemical shifts of the backbone NH signals of ^15^N-labeled H96R BOLA3 were affected by the addition of unlabeled GLRX5 (Figure 3A,B), thus indicating the occurrence of an interaction between the two proteins. The spectral changes indicated that the two proteins undergo exchange in a fast-to-intermediate regime on the NMR time scale. The affected residues, when mapped on the model structure of H96R BOLA3, result to be located in the α_2_ helix, the β_3_ strand, and their connecting loop, which contains the His96Arg mutation (Figure 3C).

Very similar effects, involving the same protein regions, were detected in the ^1^H-^15^N HSQC spectra of ^15^N-labeled WT BOLA3 recorded upon increasing additions of unlabeled GLRX5 (Figure S2 [19]), indicating that the His96-to-Arg mutation in BOLA3 does not significantly impact the interaction of BOLA3 with GLRX5.

**Figure 4:**
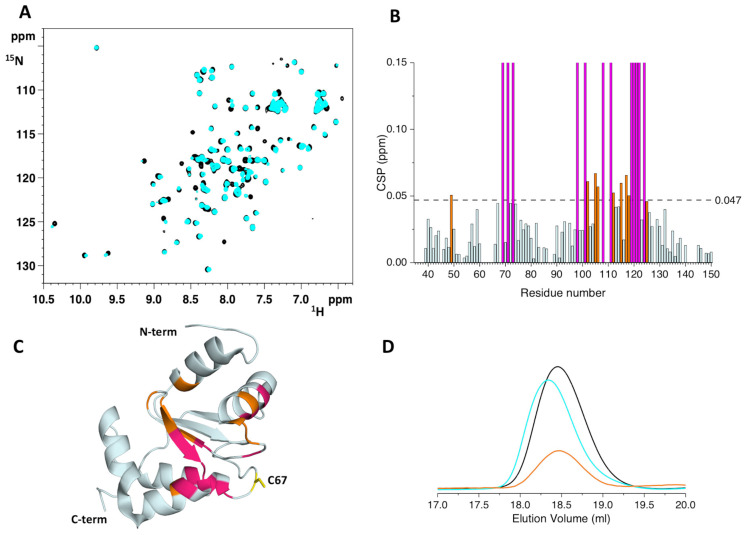
Identification of the interacting surface of apo GLRX5 with H96R BOLA3. (**A**) Superimposition of the ^1^H-^15^N HSQC spectra of apo ^15^N-labeled GLRX5 in the absence (black) and in the presence of 1.0 eq. of unlabeled H96R BOLA3 (cyan), acquired at 500 MHz and 298 K; (**B**) chemical shift perturbations of apo ^15^N-labeled GLRX5 before and after the addition of unlabeled H96R BOLA3, calculated as (((ΔH)^2^ + (ΔN/5)^2^)/2)^1/2^. The indicated threshold value was obtained by averaging CSP values plus 1σ and was used to define meaningful chemical shift differences. (**C**) Mapping of the meaningful chemical shift changes due to the interaction with H96R BOLA3 on the solution structure of apo GLRX5 (PDB: 2MMZ, [27]). Residues experiencing chemical shift variation are shown in orange, while residues experiencing line-broadening are shown in magenta in both (**B**,**C**) panels. (**D**) Analytical SEC of isolated apo GLRX5 (black line), isolated apo H96R BOLA3 (orange line) and a 1:1 mixture of apo GLRX5 and apo H96R BOLA3 (cyan line). The structure shown in panel 3C was rendered with Pymol 2.3.5.

When apo ^15^N-labeled GLRX5 was titrated with apo unlabeled H96R BOLA3, several backbone NH signals were affected also in the ^1^H-^15^N HSQC spectra of GLRX5 (Figure 4A,B). The most affected ones are those involved in the interaction of GLRX5 with GSH and those close to the Cys67 Fe-S cluster ligand (Figure 4C). The same regions of GLRX5 were affected to the same extent upon increasing additions of apo WT BOLA3 (Figure S2C, [19]), thus indicating a very similar interaction pattern for GLRX5 with either WT or H96R BOLA3.

The final 1:1 H96R BOLA3:GLRX5 mixture was also analyzed by size exclusion chromatography. The mixture eluted as a single peak at ~18.3 mL, corresponding to a species with an apparent molecular mass of ~25 kDa (Figure 4D), indicating the formation of a 1:1 heterodimeric H96R BOLA3-GLRX5 complex.

Overall, the NMR and SEC data indicate that the His96Arg mutation allows the formation of an apo heterodimeric 1:1 complex between BOLA3 and GLRX5, which does not significantly differ from the complex formed by the wild-type protein.

### 2.3. The His96Arg Substitution Does Not Impair the Formation of the BOLA3-[2Fe-2S]-GLRX5 Heterodimeric Complex

His96 in BOLA3 has been reported as a coordinating ligand of the [2Fe-2S] cluster bound to the hetero-dimeric complex formed by BOLA3 and GLRX5 [18,19]. Specifically, it has been reported that the bridged [2Fe-2S]^2+^ cluster is coordinated by the Cys59 and His96 residues of BOLA3, by the Cys67 residue of GLRX5, and by the cysteine of a glutathione (GSH) molecule [18,19]. One would expect that the cluster binding properties of the BOLA3-GLRX5 hetero-complex could be affected by the substitution of the His96 ligand of BOLA3 with an Arg residue.

To elucidate the effects of the His96Arg mutation on the cluster binding properties of BOLA3 we applied two different strategies to obtain the H96R BOLA3-[2Fe-2S]-GLRX5 hetero-complex: (i) the chemical reconstitution of a [2Fe-2S]^2+^ cluster on the apo H96R BOLA3-GLRX5 hetero-dimeric complex in the presence of FeCl_3_, Na_2_S, and GSH, and (ii) the conversion of the [2Fe-2S]-GLRX5_2_ homo-dimeric complex into the BOLA3-[2Fe-2S]-GLRX5 hetero-dimeric complex, by titrating the ^15^N-labeled [2Fe-2S]-GLRX5_2_ complex or the apo ^15^N-labeled H96R BOLA3 with apo unlabeled H96R BOLA3 or unlabeled [2Fe-2S]-GLRX5_2_ complex, respectively.

The apo H96R BOLA3-GLRX5 complex was incubated overnight with FeCl_3_ and Na_2_S in the presence of DTT and GSH. The mixture was buffer exchanged by PD-10 desalting column and then analyzed by UV-Vis and CD-Vis spectroscopies, analytical SEC, paramagnetic 1D ^1^H NMR, and continuous wave (CW) X-band EPR spectroscopy. In contrast to the WT BOLA3-GLRX5 hetero-complex, where a bridged [2Fe-2S]^2+^ cluster was easily reconstituted in vitro in the presence of GSH, FeCl_3_ and Na_2_S [18,19,21], our attempts to de novo assemble a [2Fe-2S] cluster on the H96R BOLA3-GLRX5 hetero-dimeric complex were not successful. Specifically, UV-Vis spectra showed several broad, ill-defined bands in the visible region between 300 and 700 nm (Figure S3A). In agreement, the paramagnetic 1D ^1^H NMR spectrum of the complex showed several hyperfine shifted signals in the 80–10 ppm region, that cannot be assigned to a single [2Fe-2S]-binding species, but that rather originate from a mixture of different paramagnetic species (Figure S3B). This behavior was significantly different from that of the chemically reconstituted WT BOLA3-[2Fe-2S]-GLRX5 hetero-complex [19,28], that showed well-defined UV-Vis bands at 320 nm, 400 nm, 510 nm and 590 nm and a set of broad hyperfine-shifted NMR signals between 40 and 20 ppm, and a sharper one at 14 ppm, typical of an oxidized [2Fe-2S]^2+^ cluster-containing species [19,28].

The chemically reconstituted H96R BOLA3-GLRX5 complex was EPR silent (Figure S3C, red line), and upon addition of sodium dithionite only a weak, almost axial signal appeared in the spectrum acquired at 45 K and 0.5 mW, having g values 2.02 and 1.94 (Figure S3C, black line). This signal possibly originates from an S = 1/2 spin of a protein-bound [2Fe-2S]^+^ cluster, which, however, is only present at very low concentrations.

The presence of multiple species was also detected by analytical SEC. Indeed, the chemically reconstituted H96R BOLA3-GLRX5 complex was eluted with three peaks at 17.4 mL, 17.6 mL and 18.0 mL (Figure S3D), likely arising from BOLA3/GLRX5 species with different conformations.

Overall, these data indicate that the His96 residue is required for the correct in vitro assembly of the bridged [2Fe-2S] cluster on the H96R BOLA3-GLRX5 hetero-dimeric complex and its substitution with an Arg residue does not allow the de novo formation of a single, well-defined [2Fe-2S]-binding complex, giving rise to the formation of multiple, aberrant species.

We then investigated whether the H96R BOLA3 mutant could interact with the homo-dimeric [2Fe-2S]-GLRX5_2_ complex, driving the conversion of the [2Fe-2S]-GLRX5_2_ homo-dimeric complex into the H96R BOLA3-[2Fe-2S]-GLRX5 hetero-dimeric complex [18,29,30].

**Figure 5:**
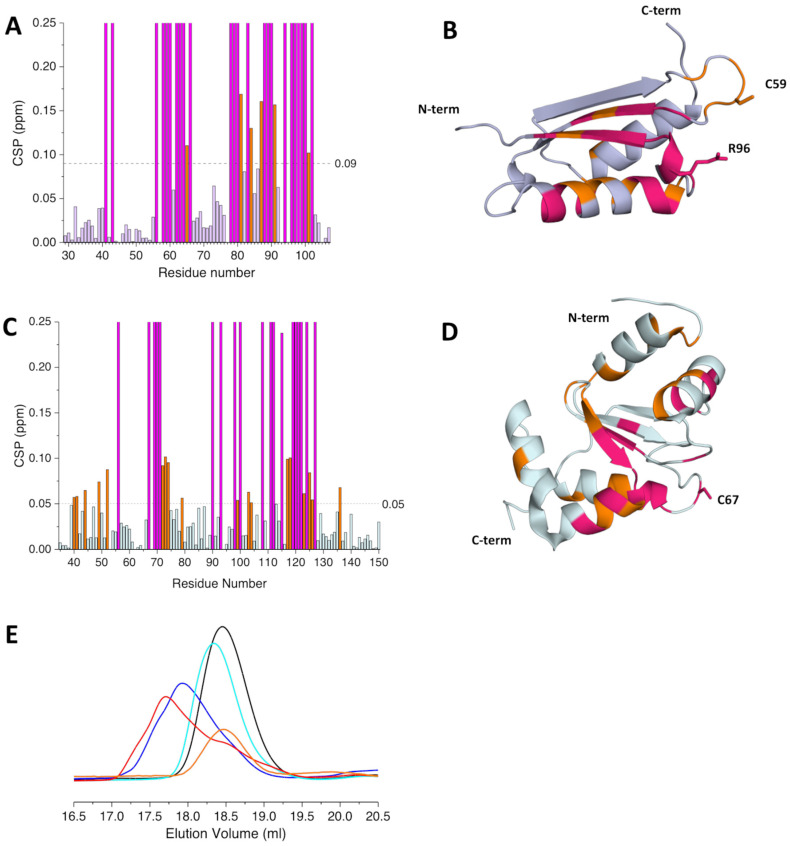
H96R BOLA3 interacts with [2Fe-2S]-GLRX5_2_. (**A**) Chemical shift perturbations of apo ^15^N-labeled H96R BOLA3 before and after the addition of 0.5 eq. of unlabeled [2Fe-2S]-GLRX5_2_, calculated as (((ΔH)^2^ + (ΔN/5)^2^)/2)^1/2^. The indicated threshold value was obtained by averaging CSP values plus 1σ and was used to define meaningful chemical shift differences. (**B**) Mapping of the meaningful chemical shift changes on the model structure of apo H96R BOLA3. Residues experiencing chemical shift variation are shown in orange, while residues experiencing line-broadening are shown in magenta in both (**B**,**C**) panels. (**C**) Chemical shift perturbations of apo ^15^N-labeled [2Fe-2S]-GLRX5_2_ before and after the addition of 2.0 eq. of unlabeled H96R BOLA3, calculated as (((ΔH)^2^ + (ΔN/5)^2^)/2)^1/2^. The indicated threshold value (obtained by averaging CSP values plus 1σ) was used to define meaningful chemical shift differences. (**D**) Mapping of the meaningful chemical shift changes on the solution structure of GSH-bound GLRX5 (PDB: 2MMZ, [27]). Residues experiencing chemical shift variation are shown in orange, while residues experiencing line-broadening are shown in magenta. (**E**) Analytical SEC of [2Fe-2S]-GLRX5_2_ before (blue line) and after (red line) the addition of 2.0 eq. of apo H96R BOLA3. The analytical SEC of isolated apo GLRX5 (black line), isolated apo H96R BOLA3 (orange line) and of a 1:1 GLRX5:H96R BOLA3 mixture (cyan line) are reported for reference. The model shown in panel 3B and 3D was rendered with Pymol 2.3.5.

Both apo ^15^N-labeled H96R BOLA3 and holo ^15^N-labeled [2Fe-2S]-GLRX5_2_ were titrated with increasing amounts of unlabeled [2Fe-2S]-GLRX5_2_ and apo H96R BOLA3, respectively. Severe line broadening was observed in the ^1^H-^15^N HSQC spectra of ^15^N-labeled H96R BOLA3 upon the addition of [2Fe-2S]-GLRX5_2_ (Figure S4A), thus indicating that the two proteins interact exchanging on an NMR intermediate time scale. Chemical shift perturbation analysis showed that, in addition to the α_2_ helix, the β_3_ strand and the loop containing the His96Arg mutation, that were also affected in the apo-apo H96R BOLA3-GLRX5 interaction, line broadening beyond signal detection was observed also for residues of the loop connecting the β1 and β2 strands, and containing Cys59 cluster ligand, and for residues subsequent to the Cys59 residue (Figure 5A,B). Furthermore, His81 and residues nearby experienced line-broadening effects in the presence of [2Fe-2S]-GLRX5_2_. However, a possible involvement of His81 in the binding of the bridged [2Fe-2S] cluster can be excluded, as this residue is located in a region of BOLA3 that is distant from the other BOLA3 Fe-S cluster ligand, i.e., Cys59. The concomitant involvement of both Cys59 and His81 in the coordination of the [2Fe-2S] cluster would imply a major conformational rearrangement of BOLA3 that is not consistent with the minor changes in the NMR spectra.

When ^15^N-labeled [2Fe-2S]-GLRX5_2_ was titrated with apo unlabeled H96R BOLA3 line broadening effects were detected also in the ^1^H-^15^N HSQC spectrum of [2Fe-2S]-GLRX5_2_ (Figure S4B). The most affected region involved the residues surrounding the Cys67 cluster ligand and the residues involved in the interaction with GSH, as observed in the apo-apo H96R BOLA3-GLRX5 complex (Figure 5B,C), indicating a very similar interacting surface.

Analytical SEC performed on the final mixtures showed a main peak eluting at 17.7 mL (Figure 5D). This elution volume is different from that of the [2Fe-2S]-GLRX5_2_ homo-dimeric complex (i.e., 17.9 mL) and from that of the apo-apo heterodimeric complex (i.e., 18.3 mL), thus indicating the conversion of the [2Fe-2S]-GLRX5_2_ homo-dimeric complex into an H96R BOLA3-[2Fe-2S]-GLRX5 heterodimeric complex. The increased elution volume of the latter complex can be attributed to a larger hydrodynamic radius, due to dynamic multi-conformational heterogeneity in solution.

**Figure 6:**
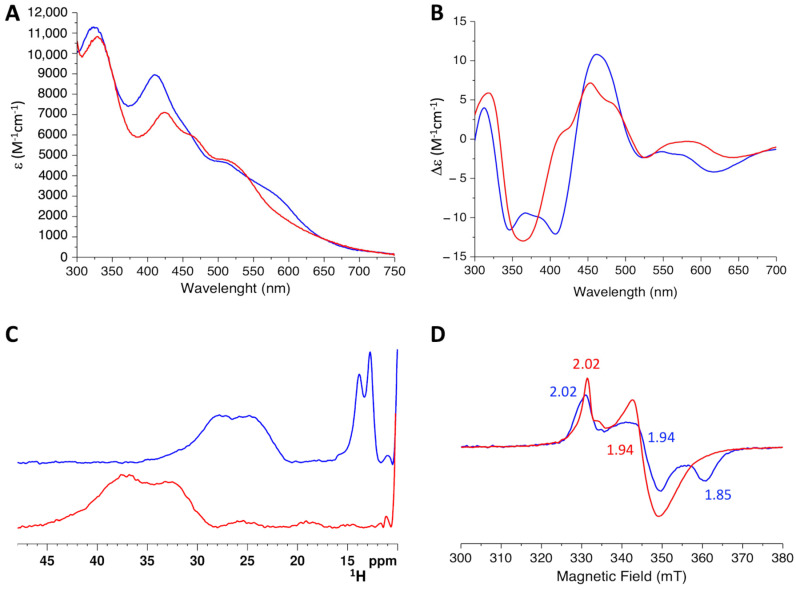
The [2Fe-2S]-GLRX5_2_ homo-dimeric complex is converted into the H96R BOLA3-[2Fe-2S]-GLRX5 heterodimeric complex. UV-Vis (**A**), CD (**B**), 1D ^1^H paramagnetic NMR spectra (**C**) of [2Fe-2S]^2+^-GLRX5_2_ before (blue) and after (red) the addition of 2.0 eq. of H96R BOLA3. (**D**) EPR spectra of dithionite reduced [2Fe-2S]^+^-GLRX5_2_ before (blue) and after (red) the addition of 2.0 eq. of H96R BOLA3, recorded at 45 K and 0.5 mW.

The UV-Vis spectrum of the new complex is characterized by well-defined absorption bands at ~330 nm, 424 nm, 460 nm, and 510 nm, which are typical of oxidized [2Fe-2S]^2+^ centers [19,29,30,31,32] (Figure 6A). In agreement, the CD-Vis spectrum showed positive bands at ~319 nm, 420 nm, 454 nm, 479 nm, and negative bands at 365 nm, 524 nm and 639 nm, which are typical of [2Fe-2S]^2+^ cluster-binding protein [18,29,30] (Figure 6B). Both spectra differ from that of the [2Fe-2S]-GLRX5_2_ complex (Figure 6A,B), in agreement with the formation of a new [2Fe-2S]-binding species.

The paramagnetic 1D ^1^H NMR spectrum of the 2:1 H96R BOLA3:[2Fe-2S]-GLRX5_2_ mixture showed broad, unresolved, hyperfine shifted signals in the 45-25 ppm region, typical of βCH_2_ protons of the residues bound to an oxidized [2Fe-2S]^2+^ cluster [33] (Figure 6C, red line). This spectrum significantly differs from that of [2Fe-2S]^2+^-GLRX5_2_ before the addition of H96R BOLA3 (Figure 6C, blue line), which showed a broad hyperfine-shifted NMR signal in the 35–20 ppm region and sharper signals at 14 and 13 ppm, which arise from the βCH_2_ and αCH protons, respectively, of the Cys67 residues bound to the [2Fe-2S]^2+^ cluster in [2Fe-2S]^2+^-GLRX5_2_ complex. The different chemical shift values observed for the two [2Fe-2S]^2+^ complexes indicate that when H96R BOLA3 is added to [2Fe-2S]^2+^-GLRX5_2_ complex, the [2Fe-2S]^2+^ cluster coordination environment changes.

CW X-band EPR spectra acquired on [2Fe-2S]^2+^-GLRX5_2_ before and after the addition of H96R BOLA3 support the previous conclusion. Indeed, both complexes were EPR silent before the addition of sodium dithionite, as expected for the S = 0 ground state of oxidized [2Fe-2S]^2+^ clusters. Upon addition of sodium dithionite, signals appeared in the EPR spectra which are different for [2Fe-2S]^2+^-GLRX5_2_ with respect to those of H96R BOLA3-[2Fe-2S]^2+^-GLRX5 complex. Specifically, the spectrum of dithionite-reduced [2Fe-2S]^+^-GLRX5_2_ acquired at 45 K and 0.5 mW showed a rhombic signal with g values 2.02, 1.94 and 1.85 (Figure 6D, blue line). The spectrum of dithionite-reduced H96R BOLA3-[2Fe-2S]^+^-GLRX5 was characterized by an almost axial signal with g values 2.02 and 1.94 (Figure 6D, red line). Both EPR spectra are due to the S = 1/2 spin of a reduced protein-bound [2Fe-2S]^+^ cluster, but the difference in the g values indicates differences in the coordination environment of the two clusters.

Moreover, the comparison of the UV-Vis, CD-Vis, paramagnetic 1D ^1^H NMR and EPR spectra of the H96R BOLA3-[2Fe-2S]^2+^-GLRX5 complex with those reported for the WT BOLA3-[2Fe-2S]^2+^-GLRX5 complex [18,19,28], clearly indicated that a different type of [2Fe-2S] cluster coordination occurs in the H96R BOLA3-[2Fe-2S]^2+^-GLRX5 complex due to the His96Arg substitution, thus confirming that the His96 residue in WT BOLA3 is involved in cluster coordination [19].

Overall, these data indicate that His96 is strictly required for the de novo assembly of the [2Fe-2S] cluster on the hetero-dimeric BOLA3-GLRX5 complex. However, it is not required for the conversion of the [2Fe-2S]-GLRX5_2_ homo-complex into the BOLA3-[2Fe-2S]-GLRX5 hetero-complex.

### 2.4. His96Arg Mutation Impairs the [2Fe-2S] Cluster Chaperone Activity of the BOLA3-[2Fe-2S]-GLRX5 Complex

In vivo and in vitro studies proposed a role for the BOLA3-[2Fe-2S]-GLRX5 hetero-complex in the maturation of [4Fe-4S] proteins [17,18,21]. Indeed, it was recently shown that the BOLA3-[2Fe-2S]-GLRX5 heterocomplex can transfer in vitro its [2Fe-2S]^2+^ cluster to apo NFU1, thus assembling a [4Fe-4S]^2+^ cluster on apo NFU1 in the presence of generic reductants, such as GSH and DTT [21]. Since [4Fe-4S] NFU1 is involved in the final steps of the maturation of mitochondrial [4Fe-4S] cluster-binding enzymes [17,22], an impairment of the [2Fe-2S]-cluster chaperone activity of the H96R BOLA3-[2Fe-2S]-GLRX5 complex could explain the phenotype associated with His-to-Arg pathogenic mutation in MMDS2 [23,24,25].

The ability of the mutated H96R BOLA3-[2Fe-2S]-GLRX5 complex to transfer its bridged [2Fe-2S]^2+^ cluster to apo NFU1 was therefore investigated by paramagnetic 1D ^1^H NMR spectroscopy and compared with that of the WT BOLA3-[2Fe-2S]-GLRX5 complex. Indeed, [4Fe-4S]^2+^ NFU1 and the H96R/WT BOLA3-[2Fe-2S]-GLRX5 heterocomplexes have distinct NMR spectra [19,21] that allow to easily address the formation of a cluster on NFU1.

**Figure 7:**
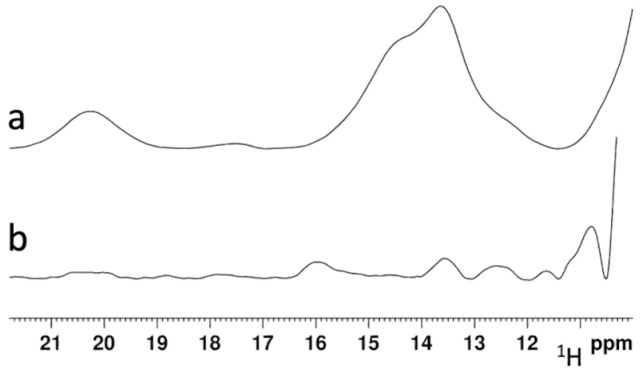
His96Arg mutation abolish the ability of the BOLA3-[2Fe-2S]-GLRX5 complex to assemble a [4Fe-4S]^2+^ cluster on NFU1. [4Fe-4S]^2+^-NFU1 hyperfine-shifted signals regions of the 1D ^1^H NMR spectra of 2:1 mixtures of WT BOLA3-[2Fe-2S]^2+^-GLRX5 (a) or H96R BOLA3-[2Fe-2S]^2+^-GLRX5 hetero-complex (b) and apo NFU1.

Apo NFU1 was incubated overnight with H96R BOLA3-[2Fe-2S]-GLRX5 or WT BOLA3-[2Fe-2S]-GLRX5 hetero-complex in a 1:2 ratio, in the presence of 5 mM DTT and 5 mM GSH, that were previously used as an electron source for the reductive coupling of the two [2Fe-2S]^2+^ clusters donated by BOLA3-[2Fe-2S]-GLRX5 to apo NFU1 [21]. These reaction conditions were the same as those previously applied [21]. Paramagnetic 1D ^1^H NMR spectra were acquired on the two reaction mixtures to monitor the formation of a [4Fe-4S]^2+^ cluster on NFU1. As expected, the NMR spectra of the WT BOLA3-[2Fe-2S]-GLRX5/NFU1 reaction mixture showed the presence of three hyperfine-shifted signals at 20.3, 14.5, and 13.6 ppm (Figure 7a), which compare well to those observed in the paramagnetic 1D ^1^H spectrum of [4Fe-4S]^2+^-NFU1 and assigned to the βCH_2_ signals of Cys residues bound to the [4Fe-4S]^2+^ cluster of NFU1 [21]. These spectra features indicate that two [2Fe-2S]^2+^ clusters are transferred from WT BOLA3-[2Fe-2S]-GLRX5 to NFU1. On the contrary, the paramagnetic 1D ^1^H NMR spectra of the H96R BOLA3-[2Fe-2S]-GLRX5/NFU1 reaction mixture, did not show any new signal corresponding to [4Fe-4S]^2+^ NFU1 (Figure 7b), thus indicating that no cluster transfer/assembly occur from the H96R BOLA3-[2Fe-2S]-GLRX5 complex to NFU1.

## 3. Discussion

Human BOLA3 is an essential ISC assembly factor, acting in the late stages of the Fe-S biosynthesis pathway, for the maturation of specific [4Fe-4S]-binding proteins [17,18,34]. BOLA3 interacts with its protein partner GLRX5 forming a [2Fe-2S] cluster-bridged hetero-dimeric complex, that can transfer, in vitro, its [2Fe-2S] cluster to apo NFU1, to form [4Fe-4S]^2+^-NFU1 in the presence of a reductant [21].

Inherited mutations in the BOLA3 gene cause severe cellular defects, which are associated with the mitochondrial human disease MMDS2 [11,14]. In addition to four non-sense mutations that abrogated the expression of full-length protein [11,34,35], four missense mutations, resulting in the substitution of a single amino acid in the BOLA3 protein, have been identified in patients with MMDS2, i.e., Cys59Tyr, Ile67Asn, His96Arg and Arg99Trp [11,14]. Among them, the Cys59Tyr and His96Arg mutations involve two highly conserved residues, identified as the coordinating ligands of the [2Fe-2S] cluster bridging BOLA3 and GLRX5 in the BOLA3-[2Fe-2S]-GLRX5 hetero-complex. The point mutation of these residues is expected to perturb the binding of the [2Fe-2S] cluster to BOLA3, and thus to affect the [2Fe-2S] cluster chaperone activity proposed for the BOLA3-GLRX5 hetero-complex [21]. However, a very different outcome was reported for patients carrying the two Cys59Tyr and His96Arg mutations. Indeed, while all the patients carrying the His96Arg mutation died within their first year of life [23,24,25], the Cys59Tyr mutation resulted in a different phenotype, with the complete recovery of the patient motor function and speech at the age of 8 [36]. Recently, the effects of the Cys59Tyr mutation on the formation of the BOLA3-GLRX5 hetero-complex and the iron-sulfur cluster-binding and -transfer properties of such complex were investigated in vitro. It was shown that the substitution of Cys59 with a Tyr residue structurally perturbs the Fe-S cluster-binding region of BOLA3. However, it still allows the formation of an aberrant C59Y BOLA3-[2Fe-2S]-GLRX5 heterocomplex [28], that can still act as a [2Fe-2S] cluster chaperone for NFU1 [28], although with decreased efficiency with respect to the WT BOLA3-[2Fe-2S]-GLRX5 complex [21,28]. These findings provided a rationale for the peculiar phenotype observed in the patient carrying the Cys59Tyr mutation [36], suggesting that the performances of the aberrant C59Y BOLA3-[2Fe-2S]-GLRX5 heterocomplex might not be sufficient in the early stages of human development, during which the functional processes performed by BOLA3 are much more critical. However, in the latest stages of human development, which are characterized by a lower requirement of BOLA3 functions, the residual activity found for C59Y BOLA3-[2Fe-2S]-GLRX5 heterocomplex can fully satisfy the BOLA3-dependent physiological processes, thus allowing a complete clinical recovery.

Conversely to the Cys59Tyr mutation of BOLA3, the His96Arg mutation led to an extremely severe phenotype, with the majority of the symptoms arising from defects in the maturation of mitochondrial [4Fe-4S] binding proteins. Specifically, all patients showed a reduced or impaired activity of mitochondrial respiratory complexes and lipoic acid-dependent enzymes, such as pyruvate dehydrogenase (PDH), 2-ketoglutarate dehydrogenase (KGDH), and protein H of the Glycine Cleavage System (GCS) [16], while no effects were reported for the cytoplasmic Fe-S proteins biogenesis. These data strongly support an alteration of the BOLA3 cellular function caused by the His96Arg mutation. Indeed, previous studies performed in yeast showed that deletion of the BOLA3 gene does not affect the activity of cytosolic [4Fe-4S] enzymes, but severely compromises mitochondrial function [18].

Since the investigation of the disease-causing BOLA3 variants can help in understanding the cellular function of BOLA3, in this work we have investigated the effects of the His96Arg mutation on the structure of BOLA3 and its interaction with its partner protein GLRX5.

His96 residue is located in the flexible loop region connecting the α2 helix and the β3 strand [19]. The residue is highly solvent-exposed and not in contact with other protein residues. When substituted by an Arg, the latter residue remained still solvent-exposed in the H96R BOLA3 model structure. As expected, our ^1^H-^15^N HSQC experiments indicated that the His96Arg mutation only perturbs residues near the mutation, without significantly affecting the overall BOLA3 protein fold. Indeed, both ^1^H-^15^N HSQC and CD spectra were indicative of a well-structured protein, having the same fold and stability as the wild-type protein. Moreover, analytical SEC experiments indicated that the His96Arg mutation does not affect the quaternary structure of BOLA3, being both the mutant and wild-type proteins monomeric, and eluting with the same elution volume, that correlate to similar structure/conformation between the two proteins.

These data suggest that the pathogenic effect of the mutation is likely not related to a decreased folding stability, and thus the His96Arg mutation is not expected to alter the cellular protein turnover with respect to the wild-type protein.

We also found that the mutation does not impair the interaction of H96R BOLA3 with its partner protein GLRX5. Indeed, the two proteins form a 1:1 hetero-dimeric complex with a comparable affinity to the WT BOLA3 protein. Chemical shift perturbation analysis indicated that the interacting surfaces between H96R BOLA3 and GLRX5 proteins are substantially conserved with respect to that found for WT BOLA3 and GLRX5 ([19] and this work).

The function of the BOLA3/GLRX5 interaction is not yet completely understood. However, it has been proposed that the hetero-dimeric complex formed by the two proteins might act as a [2Fe-2S] cluster chaperone for the maturation of specific [4Fe-4S]-binding proteins such as NFU1 [17,21]. To acquire insights on the pathogenic effects of the His96Arg variant, we investigated how the mutation impacts the formation of the H96R BOLA3-[2Fe-2S]-GLRX5 complex, and on the ability of the latter complex to assist the assembly of a [4Fe-4S]^2+^ cluster on apo NFU1. The mechanism through which the BOLA3-[2Fe-2S]-GLRX5 hetero-dimeric complex is matured in cells is still unknown. The [2Fe-2S]-GLRX5_2_ homo-dimer might be converted into the BOLA3-[2Fe-2S]-GLRX5 hetero-dimer upon interaction with apo BOLA3, or it is possible that BOLA3 and GLRX5 proteins interact in their apo forms, and then receive a [2Fe-2S] cluster from another component of the ISC machinery [6,37].

Conversely, to what obtained for the WT BOLA3, our attempts to chemically reconstitute a [2Fe-2S] cluster on the apo H96R BOLA3-GLRX5 heterocomplex did not lead to a unique [2Fe-2S] cluster-binding hetero-complex, but rather to a mixture of species with different spectroscopic properties and conformations. The observed conformational heterogeneity likely originates from cluster-ligand exchange equilibria caused by the lack of the conserved His96 ligand, which indeed plays an important role in stabilizing the [2Fe-2S] cluster binding hetero-complex in a unique conformation. On the other hand, we found that the H96R BOLA3 mutant was able to coordinate the GLRX5-bound [2Fe-2S] cluster, replacing one molecule of GLRX5 in the [2Fe-2S]-GLRX5_2_ homo-dimer, and thus converting it into the H96R BOLA3-[2Fe-2S]-GLRX5 hetero-dimeric complex.

The H96R BOLA3-[2Fe-2S]-GLRX5 hetero-complex showed significantly different NMR and EPR spectra with respect to those of the WT BOLA3-[2Fe-2S]-GLRX5 complex [19], suggesting a different coordination environment of the cluster arising from the lacking of the His96 cluster ligand. We can speculate that the different environment experienced by the [2Fe-2S] cluster in the H96R BOLA3-[2Fe-2S]-GLRX5 complex might affect the reactivity of the cluster itself thus suggesting a factor explaining the pathogenic effects of the His96Arg mutation. Indeed, we found that conversely to the wild-type complex, the H96R BOLA3-[2Fe-2S]-GLRX5 complex was not able to transfer its [2Fe-2S]^2+^ cluster and to assemble a [4Fe-4S]^2+^ cluster on NFU1. These findings are supported by data reported for the yeast homolog of BOLA3, which showed that His-to-Ala mutation of the invariant C-terminal His corresponding to His96 in human BOLA3, negatively affected respiratory growth of cells, indicating that His96 residue plays a crucial role for the cellular function of BOLA3 [17,18].

Overall, these findings suggest that the His96Arg mutation might negatively perturb the pathway involving the BOLA3-[2Fe-2S]-GLRX5 hetero-complex and NFU1 in cells, with negative consequences on the maturation of mitochondrial [4Fe-4S] cluster-containing enzymes, and thus allow to rationalize the severe MMDS2 phenotype associated with the His96Arg mutation.

Consistent with our findings showing that His96Arg mutation of BOLA3 impairs the maturation of [4Fe-4S]-NFU1, all the patients carrying the mutation displayed combined mitochondrial respiratory chain complexes deficiency, likely resulting in defects in the oxidative phosphorylation (OXPHOS) process. Indeed, possible future therapies targeting MMDS2 could be based on the development of compounds that enable mitochondria to compensate for deficient OXPHOS, bypassing for instance complex I and providing electrons directly to complex III [38].

In conclusion, our in vitro investigation of the effects of the His96Arg mutation on the structure, cluster-binding, and transfer properties of BOLA3 upon interaction with GLRX5 contributed to the understanding of the molecular basis of the His96Arg variant-dependent MMDS2.

## 4. Materials and Methods

### 4.1. Proteins Expression and Purification

The cDNA encoding for the H96R BOLA3 mutant, inserted into the pTwist ENTR vector, was acquired from Twist Bioscience (South San Francisco, CA, USA). Gateway Cloning Technology (Invitrogen, Waltham, MA, USA) was used to insert the H96R BOLA3 mutant gene into the pETG-20A vector, to obtain the N-terminal Trx-His_6_- tagged H96R BOLA3 protein. *E. coli* BL21-Gold (DE3) competent cells (Agilent Technologies, Santa Clara, CA, USA) were transformed with the obtained pETG-20A plasmid containing the H96R BOLA3 gene. Cells were grown overnight at 37 °C in 20 mL Luria-Bertani (LB) medium supplemented with ampicillin (100 μg/mL). Then cultures were diluted 1:50 in M9 minimal medium (with 1 g of (^14^NH_4_)_2_SO_4_) or (^15^NH_4_)_2_SO_4_) and 3 g of glucose) containing 100 μg/mL ampicillin, and cells were grown at 37 °C and shaken at 180 rpm, till OD_600_ = 0.6–0.8. Protein expression was induced by adding 0.5 mM isopropyl-D-1-thiogalactopyranoside (IPTG), shaking at 120 rpm for 5 h at 30 °C. Cells were harvested by centrifugation at 7500 rpm for 15 min and then resuspended in 50 mM phosphate buffer pH 8.0, 300 mM NaCl, 10 mM imidazole (binding buffer, BB from now on), supplemented with protease inhibitors (Roche, Penzberg, Germany), 3 mM DTT, lysozyme (0.25 mg/mL), DNase (12.5 μg/mL) and 1 mM MgSO_4_, and then lysed by sonication (30 s for 10 times, 3 min resting). The lysate was centrifugated by ultra-centrifuge at 35,000 rpm for 45 min. All the following purification steps were performed under aerobic conditions. The soluble extract was loaded on a HisTrap HP column (Cytiva, Marlborough, MA, USA) and the Trx-His_6_-tagged protein was eluted with a 50 mM phosphate buffer pH 8.0, 300 mM NaCl, 500 mM imidazole (elution buffer, EB from now on). The protein was then buffer-exchanged in BB. The cleavage of the Trx-His_6_-tag was performed by incubation overnight, at room temperature with tobacco etch virus (TEV) protease in the presence of 3 mM DTT. The separation of the cleaved H96R BOLA3 protein from the Trx-His_6_-tag was performed by a HisTrap HP column (Cytiva, Marlborough, MA, USA). The protein was then purified by Size Exclusion Chromatography on a Superdex 75 10/60 column (Cytiva, Marlborough, MA, USA) in 50 mM phosphate buffer pH 7.0, 150 mM NaCl. H96R BOLA3 protein was obtained with a final yield of 23 mg per liter of M9 culture.

The expression and purification of WT BOLA3, apo GLRX5, [2Fe-2S]-GLRX5_2_ and apo NFU1 were obtained following previously reported protocols [18,21,27].

The buffer was exchanged by the PD-10 desalting column when needed for the biochemical and spectroscopic characterization of the proteins. The purity grade of the proteins was assessed by SDS-PAGE electrophoresis visualized with Coomassie Blue stain (Figure S5).

### 4.2. In Vitro Chemical Reconstitution of Apo Complex

The apo H96R BOLA3-GLRX5 complex, obtained by ^1^H-^15^N HSQC NMR titration, was chemically reconstituted in vitro in anaerobic conditions, working inside an anaerobic chamber (O_2_ < 1 ppm), and by previously degassing the buffers. The chemical reconstitution of the Fe-S cluster was performed starting from a hetero-complex concentration of 60 μM, in 50 mM Tris∙HCl pH 8.0, 100 mM NaCl, 5 mM DTT and 5 mM GSH buffer with fourfold excess of FeCl_3_ and Na_2_S, for 16 h at room temperature. PD-10 desalting column (Cytiva, Marlborough, MA, USA) was performed to remove the excess of FeCl_3_ and Na_2_S from the reaction mixture.

Chemical reconstitution of WT BOLA3-[2Fe-2S]-GLRX5 was performed by following a previously reported procedure [18,19].

### 4.3. Biochemical and Spectroscopic UV-Vis, NMR and EPR Methods

The quaternary structure of the proteins was analyzed through analytical SEC on a Superdex 200 10/300 Increase column (Cytiva, Marlborough, MA, USA). The column was calibrated with a gel filtration marker calibration kit, 12.4–2000 kDa (Sigma-Aldrich, Saint Louis, MO, USA), to obtain the apparent molecular masses of the detected species. Samples were loaded on the pre-equilibrated column with degassed 50 mM sodium phosphate buffer pH 7.0, NaCl 200 mM, 5 mM GSH, and 5 mM DTT. Elution profiles were recorded at 280 nm with a flow rate of 0.7 mL/min. Protein concentrations were in the 0.05–0.1 mM range.

UV-Vis spectra were acquired anaerobically on a Cary 50 Eclipse spectrophotometer at room temperature, in 50 mM phosphate buffer pH 7.0, 150 mM NaCl, 5 mM GSH, and 5 mM DTT.

CD spectra were acquired anaerobically on a JASCO J-810 spectropolarimeter in 50 mM phosphate buffer pH 7.0, 150 mM NaCl, 5 mM GSH, 5 mM DTT. A 1 cm path length cuvette was used for the analysis in the visible region of the spectra.

Variable temperature CD (VTCD) characterization was carried out on a JASCO J-810 spectropolarimeter, equipped with a quartz cuvette with a 0.1 cm path length. The spectra were recorded at variable temperatures in the 20 °C to 90 °C temperature range.

The structure of the H96R BOLA3 was obtained with Modeller 9.2 (https://salilab.org/modeller/) [39], using the NMR solution structure of BOLA3 as a template. The H96R BOLA3 structure was then energy minimized in explicit water using an AMBER 12.0 molecular dynamics program.

Diamagnetic 1D ^1^H and 2D ^1^H-^15^N HSQC NMR experiments were performed on ^15^N-labeled H96R or WT BOLA3 in 50 mM phosphate buffer pH 7.0, 150 mM NaCl, 5 mM DTT, 5 mM GSH, and 10% (*v*/*v*) D_2_O. The NMR spectra were acquired on Bruker AVANCE 500, 900, and 950 MHz spectrometers (Bruker, Billerica, MA, USA, at 298 K. Spectra were processed using TopSpin (Bruker BioSpin, Billerica, MA, USA) and analyzed with CARA 1.9.1 software (cara.nmr.ch). The backbone chemical shift perturbations (CSP) were calculated through the equation CSP = (((ΔH)^2^ + (ΔN/5)^2^)/2)^1/2^.

Paramagnetic 1D ^1^H NMR experiments were performed on a Bruker Avance spectrometer operating at 400 MHz ^1^H Larmor frequency and equipped with a ^1^H optimized 5 mm probe. The water signal was suppressed via fast repetition experiments and water-selective irradiation [40]. Experiments were typically performed using an acquisition time of 50 ms, and an overall recycle delay of 80 ms. Sample concentration was in the range 0.2–0.4 mM, in degassed 50 mM Tris HCl buffer pH 8.0 or phosphate buffer pH 7, 150 mM NaCl, 100% D_2_O. Squared cosine and exponential multiplications were applied prior to the Fourier Transformation. Manual baseline correction was performed using polynomial functions.

CW EPR spectra were recorded after the anaerobic reduction of the protein by the addition of 1 eq of sodium dithionite. Protein concentration was in the 0.05–0.1 mM range, in degassed 50 mM Tris HCl buffer pH 8.0, 150 mM NaCl, 5 mM GSH, 5 mM DTT, and 10% glycerol. EPR spectra were acquired at 45 K, using a Bruker Elexsys 580 spectrometer working at a microwave frequency of ca. 9.36 GHz, equipped with an SHQ cavity and a continuous flow He cryostat (ESR900, Oxford instruments, Abingdon, Oxfordshire, UK) for temperature control. Acquisition parameters were as follows: microwave frequency, 9.36 GHz; microwave power, 0.5 mW at 45 K; modulation frequency, 100 kHz; modulation amplitude, 10 G; acquisition time constant, 163.84 ms; the number of points, 1024; the number of scans, 4; field range, 2000–4000 G.

## Data Availability

The data presented in this study are available within the article text, figures, and Supplementary Materials.

## Supplementary Materials

The following supporting information can be downloaded at: https://www.mdpi.com/article/10.3390/ijms241411734/s1. References [19,27,41] are cited in the Supplementary Materials.
