# Supplementary material for: Understanding the Molecular Basis of the Multiple Mitochondrial Dysfunctions Syndrome 2: The Disease-Causing His96Arg Mutation of BOLA3
Source: Int J Mol Sci. 2023 Jul 21;24(14):11734. doi: 10.3390/ijms241411734 (PMC10380394; doi:10.3390/ijms241411734)
Supplement: Supplementary file 1 [file ijms-24-11734-s001.zip › ijms-2482967-supplementary.pdf]

Supplementary Material
for

Understanding the Molecular Basis of the Multiple Mitochondrial Dysfunctions Syndrome 2: The Disease-Causing His96Arg Mutation of BOLA3

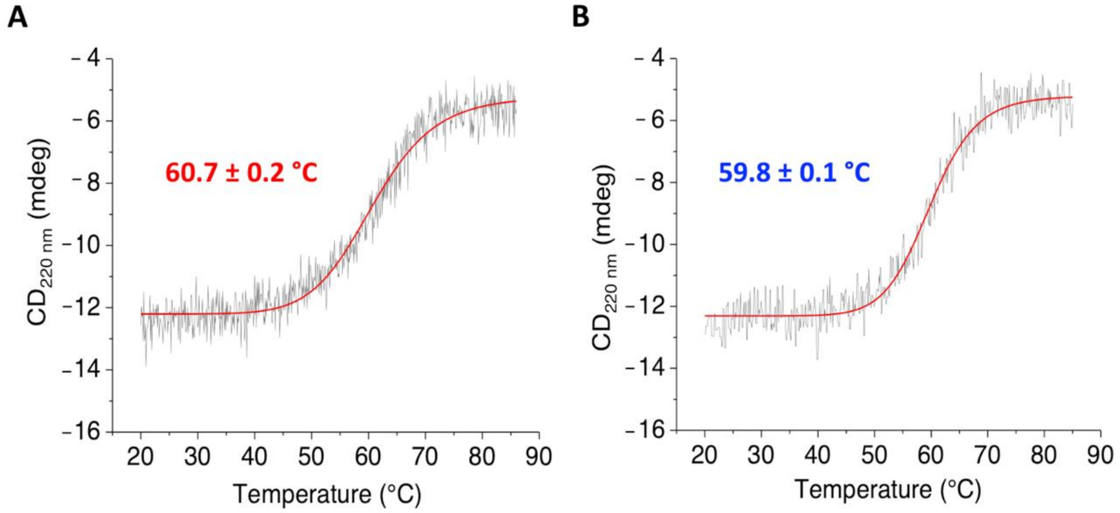

Figure S1. The His96Arg mutation does not affect the stability of BOLA3. VTCD traces for the melting of 10 μM H96R BOLA3 (A) and WT BOLA3 (B) in 50 mM phosphate, pH 7.0. Data representing change of CD at 220 nm as a function of temperature, were fit to a two-states model to obtain melting temperature values [1].

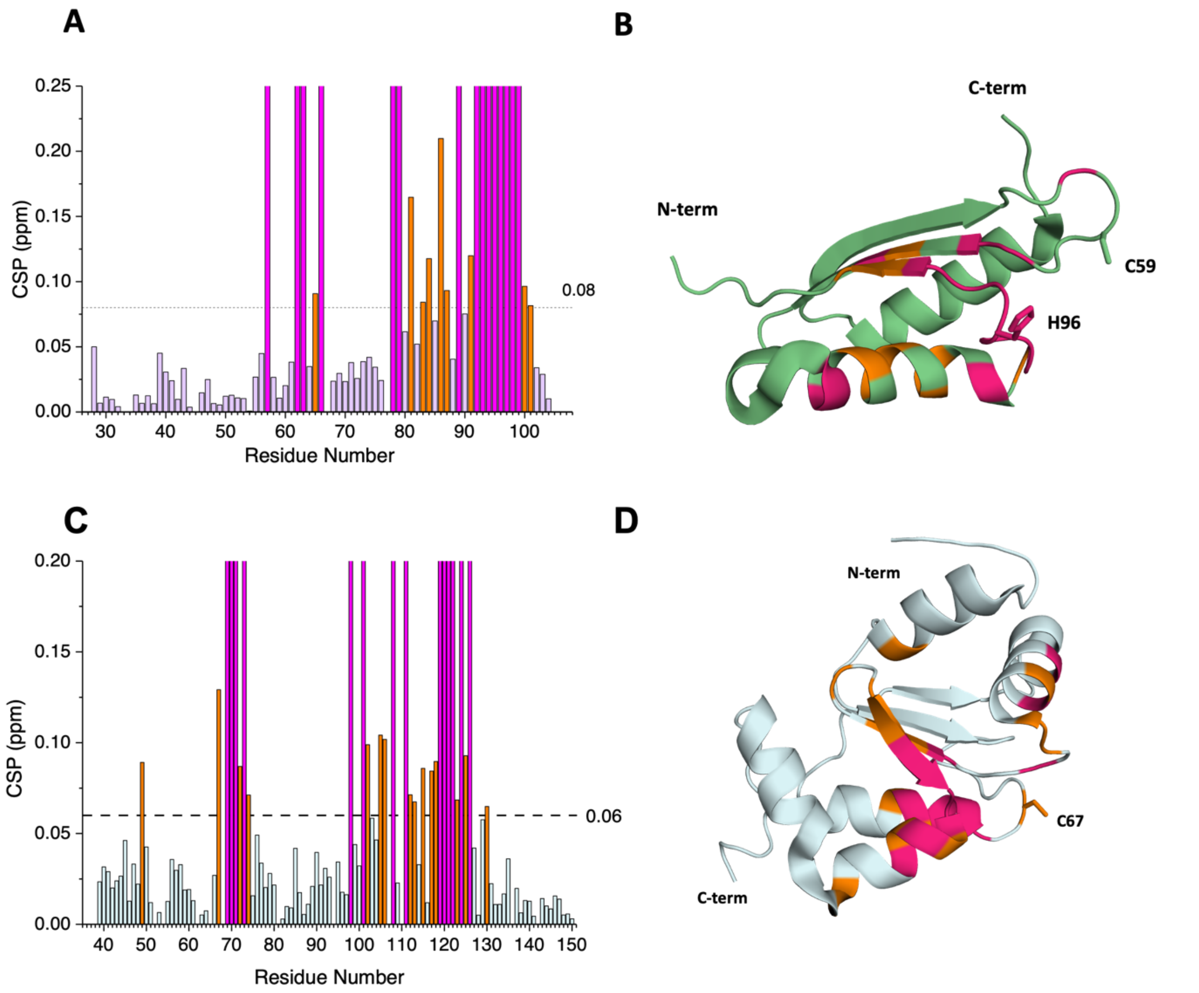

Figure S2. Mapping the interaction of WT-BOLA3 with apo GLRX5. A) Chemical shift perturbations of ^{15}N-labeled WT BOLA3 before and after the addition of 1.0 eq. of unlabeled GLRX5, calculated as $(((\Delta H)^2 + (\Delta N/5)^2)/2)^{1/2}$). The indicated threshold value (obtained by averaging CSP values plus 1σ) were used to define meaningful chemical shift differences. B) Mapping of the meaningful chemical shift changes due to the interaction with apo GLRX5 on the solution structure of WT BOLA3 (PDB: 2NCL, [2]). C) Chemical shift perturbations of ^{15}N-labeled GLRX5 before and after the addition of 1 eq. of unlabeled WT BOLA3, calculated as $(((\Delta H)^2 + (\Delta N/5)^2)/2)^{1/2}$). The indicated threshold value (obtained by averaging CSP values plus 1σ) were used to define meaningful chemical shift differences. D) Mapping of the meaningful chemical shift changes due to the interaction with WT BOLA3 on the solution structure of apo GLRX5 (PDB: 2MMZ, [3]). Residues experiencing chemical shift variation are shown in orange, while residues experiencing line-broadening are shown in magenta in all panels. The structures shown in panel 3C and 3D were rendered with Pymol 2.3.5.

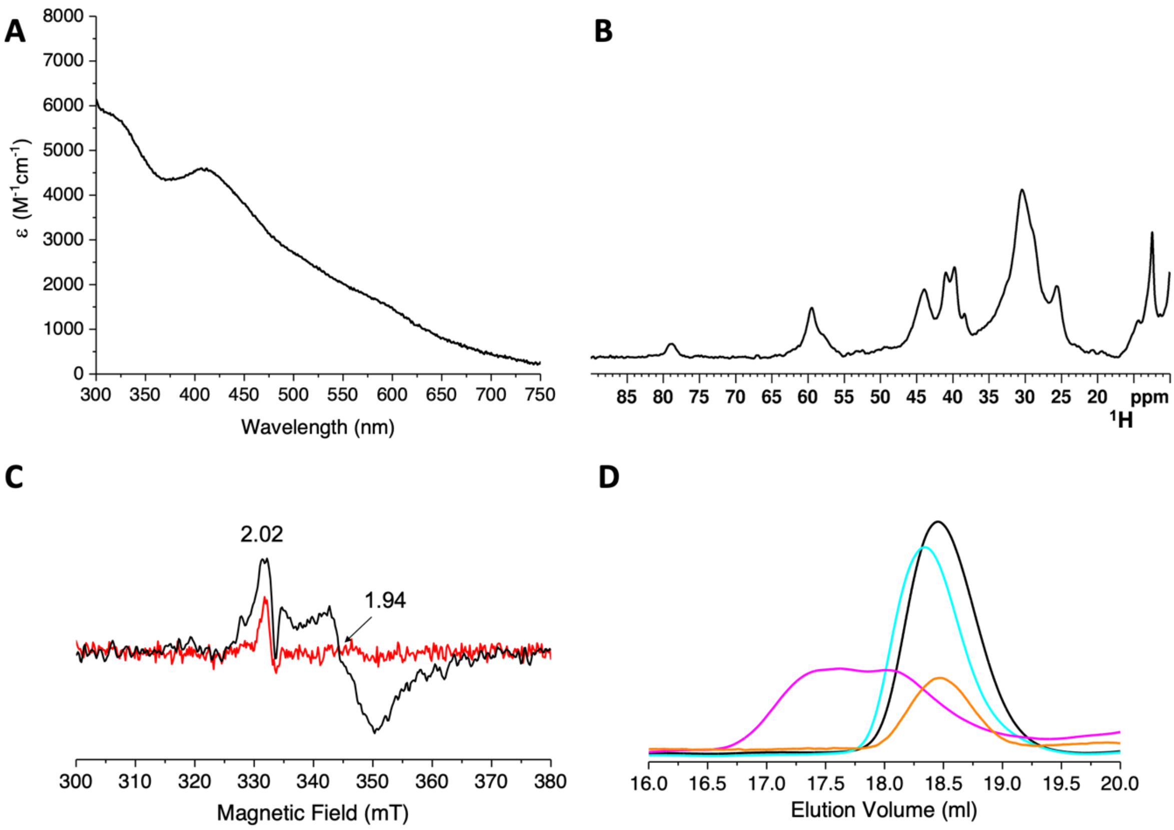

Figure S3. His96 is required for the *de novo* assembly of a [2Fe-2S] cluster on the BOLA3-GLRX5 heterocomplex. UV-Vis (**A**) and 1D ^{1}H paramagnetic NMR spectrum (**B**) of the H96R BOLA3-GLRX5 heterodimeric complex after chemical reconstitution; **C**) EPR spectra of the H96R BOLA3-GLRX5 heterodimeric complex after chemical reconstitution, before (red line) and after (black line) the addition of 1.0 eq of sodium dithionite; **D**) analytical SEC of the H96R BOLA3-GLRX5 heterodimeric complex before (cyan line) and after chemical reconstitution (magenta line). The analytical SEC curves of apo GLRX5 (black line) and apo H96R BOLA3 (orange line) are reported for reference.

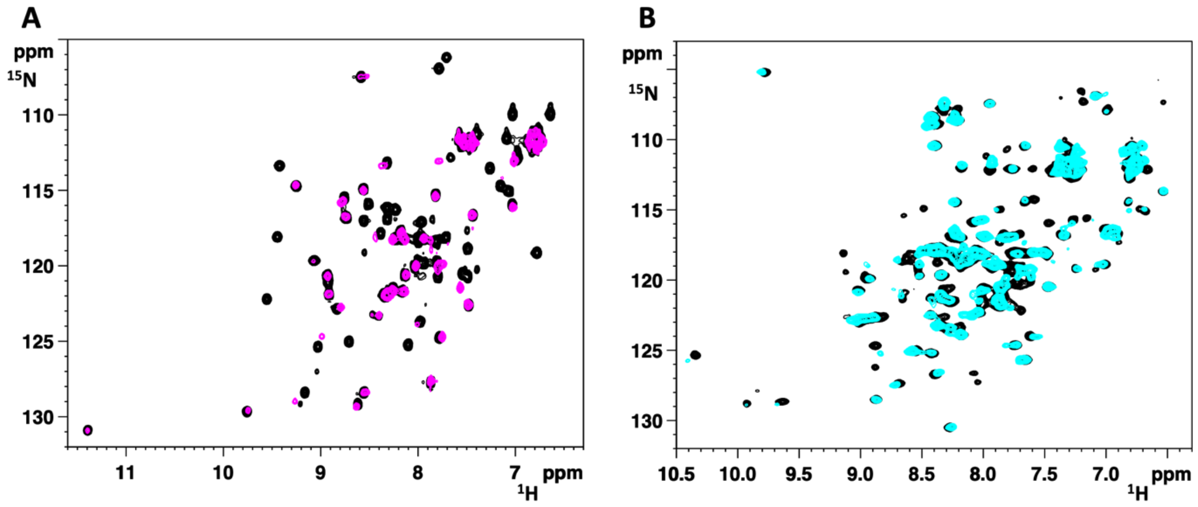

Figure S4. H96R BOLA3 interacts with [2Fe-2S]-GLRX5$_2$. A) Superimposition of the ^{1}H-^{15}N HSQC spectra of ^{15}N-labeled H96R BOLA3 in the absence (black) and in the presence (magenta) of 0.5 eq. of unlabeled [2Fe-2S]-GLRX5$_2$, acquired at 950 MHz and 298 K; B) Superimposition of the ^{1}H-^{15}N HSQC spectra of ^{15}N-labeled [2Fe-2S]-GLRX5$_2$ in the absence (black) and in the presence (cyan) of 2.0 eq. of unlabeled H96R BOLA3, acquired at 950 MHz and 298 K.

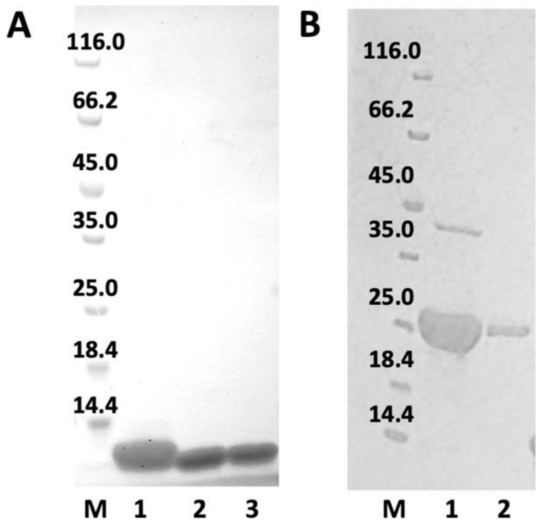

Figure S5. SDS-PAGE analysis of the recombinant proteins used in this work. A) marker (M), holo GLRX5 (lane 1), H96R BOLA3 (lane 2), WT BOLA3 (lane 3) after SEC. B) Marker (M), apo NFU1 before (lane 1) and after (lane 2) SEC. The details of the purification procedure are reported in the Materials and Methods section.

References

1. Greenfield, N.J. Using Circular Dichroism Collected as a Function of Temperature to Determine the Thermodynamics of Protein Unfolding and Binding Interactions. *Nat Protoc* **2006**, *1*, 2527–2535, doi:10.1038/nprot.2006.204.
2. Nasta, V.; Giachetti, A.; Ciofi-Baffoni, S.; Banci, L. Structural Insights into the Molecular Function of Human [2Fe-2S] BOLA1-GRX5 and [2Fe-2S] BOLA3-GRX5 Complexes. *Biochimica Et Biophysica Acta* **2017**, *1861*, 2119–2131, doi:10.1016/j.bbagen.2017.05.005.
3. Banci, L.; Brancaccio, D.; Ciofi-Baffoni, S.; Del Conte, R.; Gadepalli, R.; Mikolajczyk, M.; Neri, S.; Piccioli, M.; Winkelmann, J. [2Fe-2S] Cluster Transfer in Iron-Sulfur Protein Biogenesis. *Proceedings of the National Academy of Sciences of the United States of America* **2014**, *111*, 6203–6208, doi:10.1073/pnas.1400102111.
